# Supports and Interventions for Carers of a Person with Depressive or Anxiety Symptomology: A Systematic Review

**DOI:** 10.5964/ejop.6407

**Published:** 2022-11-30

**Authors:** Elloyse Fitzgeraldson, Frances Kay-Lambkin, Natasha Harding, Kimie M. McNaughton, Zoi Triandafilidis, Jacinta Heath, Bronte Lyford, Janine Charnley, Sally Fitzpatrick

**Affiliations:** 1 University of Newcastle, Newcastle, Australia; 2 Everymind, Newcastle, Australia; 3 Centre for Emotional Health, Macquarie University, Sydney, Australia; 4 NHMRC Centre for Research Excellence in Mental Health & Substance Abuse, Macquarie University, Sydney, Australia; 5 Hunter Medical Research Institute, Newcastle, Australia; 6 Centre for Brain and Mental Health Priority Research Centre, University of Newcastle, Newcastle, Australia; 7 Society for Mental Health Research, Victoria, Australia; 8 International Society for Research on Internet Interventions, Sanford, NC, USA; 9 Child and Adolescent Mental Health Service, Hunter New England Health, Sydney, Australia; Creighton University, Omaha, NE, USA

**Keywords:** carers, caregivers, family, anxiety, depression, mental health

## Abstract

An increasing body of research attests to the capacity of evidence-based interventions to improve outcomes for informal carers. A review of suitable supports and interventions for carers of a person with depressive or anxiety symptomology is timely. This systematic review explores intervention suitability evidence for this carer group. Searches for relevant primary studies were conducted in six databases across a 15-year timeframe (October 2004–October 2019). Studies were assessed and compared narratively and thematically. Qualitative themes were synthesised with quantitative studies to explore the extent to which carer preferences were embedded in interventions. The initial literature search produced 13,183 studies. Six studies—three randomised controlled trials (RCTs) and three mixed-method studies—were included following a double-blinded screening process, a review of reference lists and risk of bias assessment. Included studies contributed either intervention efficacy or acceptability evidence. The synthesis of qualitative themes with quantitative studies found that carer-specific needs and targeted psychoeducation were featured in interventions from all six quantitative studies. Current evidence for appropriate supports and interventions for this carer group is limited. The review uncovers a lack of interventions for carers of a person with anxiety symptomology and limited intervention suitability evidence for carers of a person with depressive symptomology. More research is needed to explore the needs and preferences of this carer group, and how best to support them.

Carers have become a strong focus in the literature and service delivery in recent years. A carer, also referred to as an informal carer, is a person, such as a partner, family member, friend, or significant other, who provides unpaid support to someone with ‘a diminishing physical ability, a debilitating cognitive condition or a chronic life-limiting illness’ (International Alliance of Carer Organizations, 2021). There are millions of carers globally, with an estimated 43.5 million carers in the United States (NAC & AARP Public Policy Institute, 2015), 7.8 million in Canada (Statistics Canada, 2018), 6.5 million in the United Kingdom (New Policy Institute, 2016) and 2.8 million in Australia (Carers Australia & Deloitte Access Economics, 2020).

Carers make a significant contribution to economies and service sectors worldwide by providing support to families and friends who would otherwise rely on formal services. Countries save between $20bn and $470bn USD per year through unpaid carer contribution (Carers Australia & Deloitte Access Economics, 2020; Carers UK & University of Leeds, 2011; Hollander et al., 2009; NAC & AARP Public Policy Institute, 2015). Carers also contribute to the community by providing practical and emotional support to their partners, family members, and friends.

Though there are positive aspects to this role, caring can be detrimental to a person’s health and wellbeing. It is well documented in the extant literature that many carers will experience financial, career, family, and social strains due to their caring role (Harris et al., 2015; Poon et al., 2017). While services and supplements exist for carers, such as psychology services and financial aid, research shows that carers of a person with a mental health condition rarely access available supports (Yesufu-Udechuku et al., 2015). In Australia, for instance, there are approximately 329,000 carers of a person with a mental illness. Yet, only a portion (34.4 percent) of these individuals access professional services, with more than a third unaware that relevant services exist (Diminic et al., 2019). This finding suggests that current interventions and supports are not sufficiently targeting mental health carers' diverse needs.

The research literature rarely refers to mental health carers of a person with depressive or anxiety symptomology, despite evidence of high prevalence rates for depression and anxiety. Data suggests that one million Australians will experience depression and two million will experience anxiety in any one year (Australian Bureau of Statistics, 2008). It is also common for individuals to present with co-occurring symptoms of depression and anxiety (Cairney et al., 2008; Rivas-Vazquez et al., 2004), or symptoms occurring alongside other physical health conditions, chronic illnesses or disabilities, such as a cancer diagnosis, diabetes and traumatic brain injury (Chireh et al., 2019; Nefs et al., 2019; Osborn et al., 2017; Williams & Dale, 2006).

Due to the highly variable and comorbid nature of depressive and anxiety symptomology, service professionals overlook many individuals experiencing symptoms of these conditions (Rivas-Vazquez et al., 2004). The fact that many people with depressive and anxiety symptoms will not meet the formal diagnosis threshold (Rivas-Vazquez et al., 2004) partly explains this finding. Yet evidence suggests that even sub-threshold depressive and anxiety symptomology can cause significant psychological impairment (Haller et al., 2014; Rodríguez et al., 2012). Without professional support, it is often the spouses, families and friends of these individuals who become the ‘carers’.

Research suggests that carers of a person with depression experience common lifestyle challenges, such as financial difficulties, feelings of distress, sleeplessness, relationship ambivalence and less engagement in social activities (Skundberg-Kletthagen et al., 2014). Expressing worry about the future, stigma and accessibility to treatment are also frequently cited challenges for carers (Highet et al., 2004; Jeglic et al., 2005; Lemmens et al., 2009). However, less is known about the specific needs of carers of a person with anxiety. The experiences of those supporting someone with symptoms of these conditions without a formal diagnosis are also rarely considered in current policy and service delivery.

By systematically identifying and comparing studies on supports and interventions for carers of a person with depressive or anxiety symptomology, the following review identifies current evidence for intervention suitability for this carer group. The review undertakes three methods of analysis. First, a synthesis of available efficacy and feasibility studies seeks to identify existing quantitative evidence for intervention suitability. Next, a thematic analysis of included qualitative studies seeks to summarise existing qualitative evidence for intervention suitability. Finally, a synthesis of qualitative themes with quantitative studies explores whether current evidence-based interventions reflect carers' needs and preferences expressed in the thematic analysis.

## Method

### Inclusion and Exclusion Criteria

Studies were eligible for inclusion if they described supports and interventions targeting carers aged 16 years and over; included carers supporting a person with depression and/or anxiety symptomology; reported their results for carer participants separate to any other participants (such as care recipients); had a publication in a peer-reviewed journal and reported their study in English. Studies that did not provide strategies for carers exclusively or that included carers under 16 years of age were excluded. Studies that referred to some recipients of care with symptoms of depression or anxiety, but not all participants in the sample, were also excluded.

### Data Sources and Search Strategies

A systematic search of six databases was conducted: PsycINFO, MEDLINE, Embase, Psychology and Behavioural Sciences, Psychology Database and Scopus. The search was limited to human studies only, written in English and spanned a 15-year time limit, from October 30, 2019 (search commencement date) to October 30, 2004). Broad and general search terms were used to capture all potentially eligible studies including the heterogeneous nature of carers, such as family, mother, father, parent, peer, friend, significant other, partner and sibling. A search strategy example for MEDLINE is displayed in Table 1.

**Table 1:** Search Strategy Using Medline 2004–Present (October 30, 2019)

Number	Searches	Results
1	carer.mp.	4889
2	caregiver.mp.	27645
3	informal caregiver.mp.	598
4	primary caregiver.mp.	1267
5	Family/	75388
6	mother.mp.	120169
7	father.mp.	25949
8	parent.mp.	157432
9	friend.mp.	14940
10	peer.mp.	84696
11	significant other.mp.	1271
12	partner.mp.	73967
13	sibling.mp.	23107
14	1 or 2 or 3 or 4 or 5 or 6 or 7 or 8 or 9 or 10 or 11 or 12 or 13	553220
15	intervention.mp.	611135
16	support program.mp.	1561
17	Education/	20745
18	psychoeducation.mp.	2940
19	psychological.mp.	562707
20	behaviour management.mp.	255
21	behavior management.mp.	808
22	behaviour modification.mp.	574
23	behavior modification.mp.	2250
24	skills training.mp.	6465
25	skills building.mp.	315
26	cognitive behaviour therapy.mp.	1688
27	cognitive behavior therapy.mp.	2466
28	CBT.mp.	10868
29	motivational interviewing.mp.	4452
30	Social Support/	69981
31	15 or 16 or 17 or 18 or 19 or 20 or 21 or 22 or 23 or 24 or 25 or 26 or 27 or 28 or 29 or 30	1206650
32	Depression/	117414
33	Depressive Disorder/	72542
34	symptoms of depression.mp.	11836
35	Anxiety/	79956
36	anxiety disorder.mp.	15312
37	symptoms of anxiety.mp.	6341
38	32 or 33 or 34 or 35 or 36 or 37	251338
39	14 and 31 and 38	10678
40	limit 39 to (English language and yr="2004 - 2019")	7528

### Quality Assessment

Reviewers (EF and NH) used tools from the Critical Appraisal Skills Programme (2018) to assess the quality of included studies. A numerical score was awarded to each study for each question on the tool, depending on how well the study responded to the screening questions (0 = no, 1 = partly, 2 = yes; see Farrance et al., 2016). Using the CASP Randomised Controlled Trials Checklist, RCTs could score a maximum of 22 points. Non-randomised studies could score 24 on the CASP Cohort Study Checklist and Qualitative studies could score a maximum of 20 points on the CASP Qualitative Studies Checklist.

The reviewers removed two non-randomised studies from the review, based on low scores of 8/24 (Katsuki et al., 2011) and 12/24 (Bernhard et al., 2006). The studies aimed to assess intervention efficacy but did not include a control group. If the studies reported on the interventions’ feasibility and acceptability, then the study designs may have been sufficiently robust to answer these questions and pass the quality assessment.

### Data Extraction

The lead reviewer (EF) extracted data from studies using data extraction forms. Studies reporting more than one method contributed information to all applicable data extraction forms. The extracted data included the study type, study design, study population and participant demographics, type of intervention, details of reported study outcomes, details of study efficacy or acceptability to participants, risk of bias score and study limitations. Two reviewers (EF and NH) identified and resolved any discrepancies resulting from the data extraction process.

### Data Analysis

The review methodology was adapted from the Evidence for Policy and Practice Information and Co-ordinating Centre (EPPI-Centre; Farrance, et al., 2016; Thomas et al., 2004). This method addresses broad research questions where relevant qualitative and quantitative evidence exists (Clement et al., 2015). Following this approach, qualitative and quantitative studies were synthesised separately.

For the qualitative studies, thematic analysis (Braun & Clarke, 2012) was used to combine carers’ responses. The lead reviewer (EF) copied the results section of qualitative studies verbatim in Qualitative Data Analysis Software (NVivo; QSR International, 2020). To analyse the data, the reviewer systematically assigned labels or 'codes' to text (Thomas & Harden, 2008). The analysis aimed to summarise carers’ experiences engaging with interventions.

For the quantitative studies, data relating to intervention efficacy and feasibility was analysed descriptively. This approach was used in place of a meta-analysis to address the expected methodological and clinical heterogeneity across studies (Popay et al., 2006). This analysis aimed to identify the suitability of interventions for the carer group, based on quantitative data. Data was included from randomised and non-randomised studies.

Following the separate quantitative and qualitative data syntheses, the reviewer created a table to compare the qualitative themes with the quantitative studies. The table enabled the primary reviewer to assess the extent to which the intervention designs and measures used in the quantitative studies reflected the concepts identified in the qualitative analysis (Thomas & Harden, 2008).

## Results

**Figure 1:**
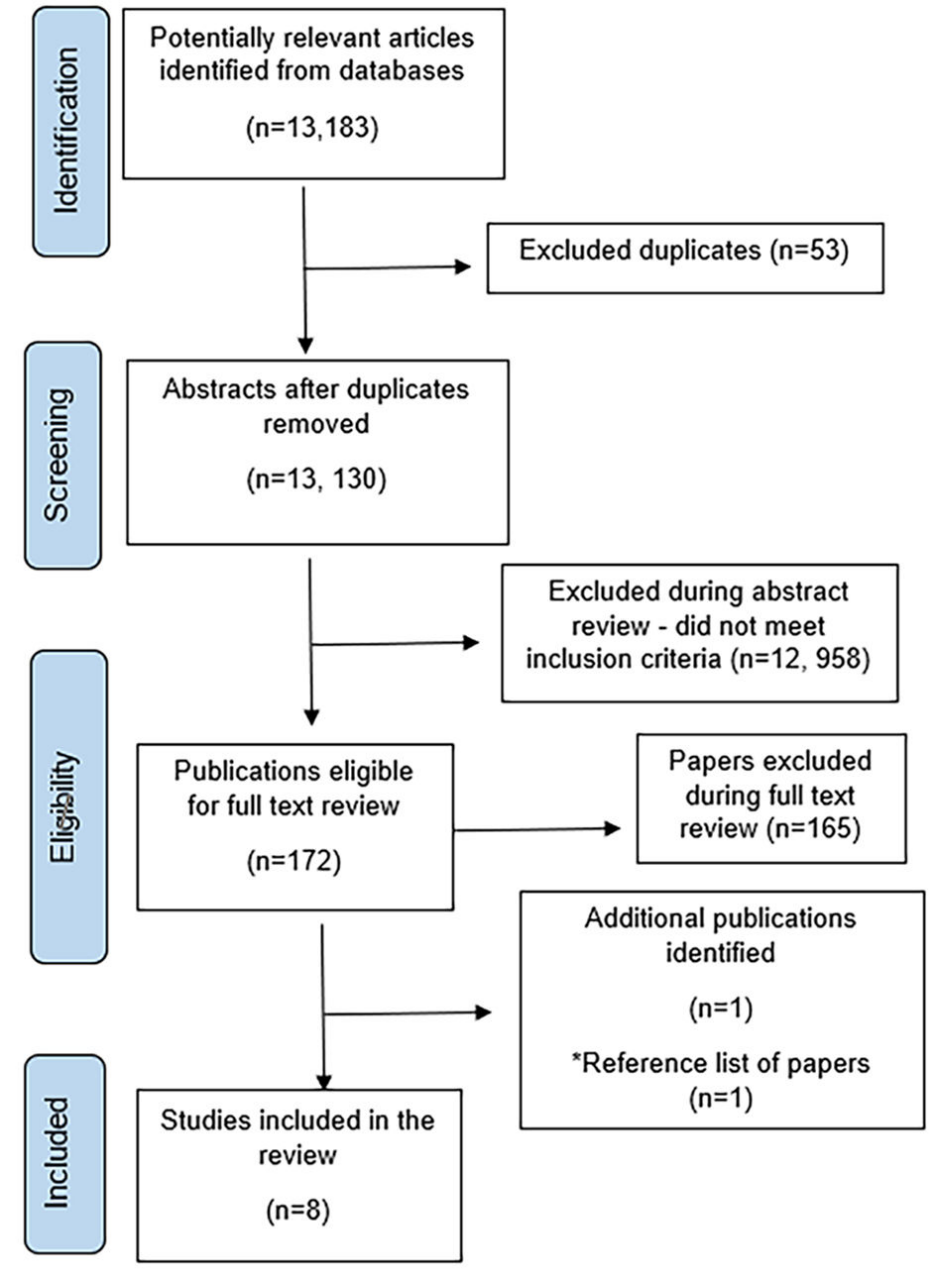
Flow Chart of Included Studies

The initial literature search produced 13,183 studies. Following abstract screening against inclusion and exclusion criteria, 172 articles progressed to the full-text review and seven studies were included (11 articles). One additional study was retrieved through a review of the reference lists of all included papers. After the exclusion of two studies as part of the risk of bias assessment, a total of six studies were included in the synthesis (three RCTs and three mixed-method studies). The mixed method studies had relevant qualitative and quantitative data. This process is outlined in Figure 1.

### Quality of Included Studies

The three RCTs scored at least 15 out of a possible 22 points for the quality assessment. The main limitations for this study type were small sample size, lack of blinding and specific samples that do not represent the carer group more broadly (e.g., carers of a person with bipolar disorder). The highest scoring mixed-method study was 16 out of a possible 24 points. These studies were low scoring due to the one arm study designs and small sample sizes. The qualitative studies scored well comparatively, receiving at least 17 out of a possible 20 points. Common limitations for this study type were no mention of the researcher critically examining their role and a lack of diversity within the sample.

### Quantitative Synthesis

The quantitative synthesis included data from three RCTs and three mixed-method studies. Sample sizes ranged from 16–121 carer participants (n = 298) and all participants were ≥ 18 years of age. Five studies reported on the mean age of participants (Berk et al., 2013; Hubbard et al., 2016; McCann et al., 2015; Perlick et al., 2018; Racey et al., 2018), with an average age of 49.6 years. Three studies reported age as a range, with participants falling between 20 and 68 years (McCann et al., 2015; Stjernswärd & Östman, 2011). Participants were parents (28 percent), unspecified relatives (18 percent), partners (eight percent), adult children (three percent) and friends/neighbours (one percent) of care recipients. The relation to care recipients was unknown for 42 percent of participants.

Four of the six studies included male and female participants (Hubbard et al., 2016; McCann et al., 2015; Racey et al., 2018; Stjernswärd & Östman, 2011) and two studies included female participants only (Berk et al., 2013; Perlick et al., 2018). Overall, 78 percent of participants were female, 15 percent were male, and seven percent did not provide data for this question. Studies were in Australia, Sweden, UK, Thailand, and the United States. Berk and colleagues (2013) recruited carers online from several English speaking developed countries, such as Australia, UK, USA, Canada, and others. Study periods ranged from six weeks to 15 months. Summaries and outcomes of studies are included in Table 2.

Participants engaged with three face-to-face interventions (Hubbard et al., 2016; Perlick et al., 2018; Racey et al., 2018), one take home manual (McCann et al., 2015) and one online platform (Berk et al., 2013; Stjernswärd & Östman, 2011).

Intervention efficacy was a primary outcome for all included RCTs. The studies measured carers’ mental health (Hubbard et al., 2016; Perlick et al., 2018), resilience (McCann et al., 2015), self-efficacy and psychoeducation (Hubbard et al., 2016) across time points. The three RCTs demonstrated significant results (p < .05).

The interventions used in the RCTs had some differences and similarities. For example, only one of the interventions targeted carers of a person with depression (McCann et al., 2015). The other two targeted carers of a person with bipolar (Hubbard et al., 2016; Perlick et al., 2018). While only two interventions used CBT strategies (McCann et al., 2015; Perlick et al., 2018), all three included psychoeducation and health-promoting strategies. Some examples include mental health promotion (Perlick et al., 2018), coping strategies (Hubbard et al., 2016) and social, and physical health promotion (Hubbard et al., 2016; McCann et al., 2015).

Each intervention also had unique features. For instance, the brief group psychoeducation included an action plan activity to complete and review with the person with bipolar (Hubbard et al., 2016). Conversely, the family focused treatment (FFT) used feedback from interviews with carers to inform module content on barriers to implementing self-care (Perlick et al., 2018). Furthermore, the guided self-help (GSH) manual was the only resource accessible from participants’ homes (McCann et al., 2015).

All three mixed-method studies were primarily concerned with carer acceptability of the intervention. However, one study was also concerned with care recipients (young people) and clinicians’ acceptability (Racey et al., 2018). The studies assessed acceptability through intervention usefulness (Berk et al., 2013), usability (Stjernswärd & Östman, 2011) and participant attendance (Racey et al., 2018). Participants from all three studies met acceptability criteria.

**Table 2:** Study Characteristics of Included Quantitative Studies

Study	Aims	Study type	Study quality	Population	Intervention	Comparative intervention	Measures	Results	Limitations
**Hubbard et al. (2016)**	The study aimed to evaluate the effectiveness of a brief group psychoeducational intervention for carers of individuals with bipolar disorder.	RCT.	17/22	Participants (*n*=32) were (a) aged ≥ 18 years and caring for someone with a diagnosed bipolar disorder. Recruitment: radio advertisements, local support/mental health services and university emails, in Perth, Australia.	Two 150- minute group sessions spaced one week apart.	Waitlist control.	Burden Assessment Scale (BAS; Reinhard et al., 1994), Knowledge of Bipolar Disorder Scale (Hubbard et al., 2016), Bipolar Disorder Self-Efficacy Scale (adapted from Smith et al).	Treatment group had significant reductions in caregiver burden (*p* < .001) and increases in bipolar disorder knowledge (*p* < .001) and bipolar disorder self-efficacy. Improvements maintained or increased to follow-up.	Small sample size, specific to bipolar carers.
**McCann et al. (2015)**	Effectiveness of a cognitive behaviour therapy (CBT) based guided self-help (GSH) manual in decreasing Expressed Emotion (EE) in carers of a person with depression.	RCT	19/22	Primary family carers (*n*=54) of outpatients with a diagnosis of depression. Recruitment: family carers of patients from the outpatient unit at Suan Prung Psychiatric Hospital, in Chiang Mai, Thailand.	Eight module CBT based GSH manual.	Standard outpatient department support.	The Resilience Scale (RS) (Wagnild & Young, 1993).	Significant difference in resilience scores between time points *(f (*2, 102) = 15.1, *p* <0.001), with a partial *η2* statistic of 0.228 indicating a large effect.	Small sample size, sample is lacking diversity.
**Perlick et al. (2018)**	Sustained effects of caregiver‐only adaptation of family‐focused treatment (FFT).	Pilot RCT.	18/22	Primary family caregivers (*n *= 36) of patients with a clinical diagnosis of bipolar I or bipolar II disorder. Recruitment: family members of patients referred from three mental health facilities, in New York, United States of America.	12–15 session FFT sessions with psychoeducation and CBT.	8–12-session health education (HE) intervention delivered via DVDs.	Center for Epidemiological Studies of Depression Scale (CES-D; Radloff, 1977), Mental Outcomes Studies Short-Form Health Survey (SF-36; Ware et al., 1995).	Significant improvements were observed for mental health outcomes (SF-36) from pre-post and maintained at 6-months follow-up (*p* = .02) and significant decrease in depression scores (CES-D) from pre-to post-treatment which was maintained over the 6-month period (*p* = .003).	Small sample size, bipolar carer specific, FFT had roughly twice the number of sessions as HE condition. Effect could be due to treatment dose.
**Racey et al. (2018)**	Feasibility and acceptability of Mindfulness–based cognitive behaviour therapy (MBCT) for young people, their parents, and clinicians.	Mixed-method feasibility study.	14/24	Parents (*n* = 29) of young people (aged 14–18 years) with depression were recruited within a single Child and Adolescent Mental Health Service (CAMHS) in Devon, England.	8 session manualised group intervention.	N/A (one arm study).	Beck Depression Inventory-II (Beck et al.,1996), Rumination Response Scale (Treynor et al. 2003), Self-Compassion Scale (Neff 2016), Mindful Attention Awareness Scale (Brown & Ryan, 2003), The Experiences Questionnaire Decentring Subscale (Fresco et al., 2007).	21 of the 25 young people/parents dyads who started the MBCT course attended at least six or more sessions, suggesting adherence.	One arm trial, small sample size.
**Berk et al. (2013)**	Acceptability and usefulness of online guidelines for caregivers of adults with bipolar.	Mixed-method feasibility study.	16/24	Participants (*n* = 121) were adult carers of adults with bipolar. Recruitment: study was advertised at mental health and carer organisations in various English-speaking countries, advertisements posted in doctors’ waiting rooms plus Google advertisements.	An information website for carers of a person with bipolar disorder.	N/A (one arm study).	Items for website usefulness on a four-point scale (‘Very useful’ to ‘Not useful at all), demographic questions and question around estimated time spent viewing the website.	97.4 percent of users found the intervention useful.	One arm study, specific to carers of people with bipolar.
**Stjernswärd & Östman. (2011)**	Feasibility of constructing a digitally based tool through an iterative design process in cooperation with potential users.	Mixed-methods feasibility study.	14/24	Participants (*n* = 16) were relatives of a person with depression. Recruitment: advertisement on in regional newspaper, website forum and carers of patients in psychiatric wards in south Sweden.	Online platform, including diary activity, social forum, and scales, as a basis for group discussions.	N/A (one arm study).	SUS (system usability scale).	The results show the pros and cons of using the online tools within a group.	Small sample size, one arm design and based on potential users’ reflections about the digital tool, not on actual user experiences.

### Qualitative Synthesis

A variety of data collection methods were used for studies with relevant qualitative data, including surveys (Berk et al., 2013), semi-structured interviews (Racey et al., 2018), online forum posts, a usability scale, and focus groups (Stjernswärd & Östman, 2011). Authors used content analysis (Berk et al., 2013; Stjernswärd & Östman, 2011) and thematic analysis (Racey et al., 2018) to assess carer responses. Study details are summarised in Table 3. Both positive and negative appraisals of the interventions were identified in carer feedback. The lead reviewer (EF) sorted these data extracts into possible themes regarding intervention suitability. Themes and associated extracts were checked and discussed by other authors until thematic consensus was reached. Carer feedback comprised of three central themes: carer-specific needs, targeted psychoeducation, and appropriate intervention design.

#### Carer-Specific Needs

Carers approved of interventions that targeted their specific needs. For example, carers in all three studies appreciated information that considered their lived experience and promoted their health and wellbeing.

Carers were optimistic about content that referenced their lived experience. For example, a carer who engaged with the online guidelines said they valued information that considered their perspective (e.g., ‘Information given specifically for caregivers, from our point of view’; Berk et al., 2013 p. 7). Similarly, carers who used the guidelines and social forum commented that stories of other carers helped them feel less alone in their experience (Berk et al., 2013; Stjernswärd & Östman, 2011).

Carers were also positive about interventions that promoted their health and wellbeing. For example, a carer who engaged with the MBCT resonated with the message to take care of oneself, stating, ‘[I]f you can manage yourself; you are in a better place to help them (the care-recipient)’ (Racey et al., 2018, p. 1070). Similarly, carers found that the online diary was a helpful tool that for prioritising their needs. One carer described the diary as a ‘think tank’ that supported them to take time and make ‘room for reflection’ (Stjernswärd & Östman, 2011).

#### Targeted Psychoeducation

Carers positively appraised targeted psychoeducation in all three studies. They were receptive towards interventions with information about mental illness, the needs of care recipients, and stigma. Participants who used the online guidelines for carers of a person with bipolar disorder were hopeful about learning more about this condition. For example, one carer stated that bipolar disorder is ‘not a death sentence’, with another reflecting that ‘information boosted (their) optimism’ (Berk et al., 2013, p. 7). Similarly, carers found that information on the website and social forum ignited a ‘curiosity and wish for more knowledge’ (Stjernswärd & Östman, 2011, p. 381).

Having a better understanding of the processes that maintain depression enabled carers to develop more empathy for the care recipient and a better understanding of the illness (Racey et al., 2018). A participant from the MBCT intervention reflected on the role of the course in supporting them to better understand their child. The carer stated that ‘It’s helped me understand my daughter as well and what she must be going through’ (Racey et al., 2018, p. 1070).

Carers also positively appraised information that addressed stigma. In one study, carers of a person with bipolar appreciated the discussion of stigmatised topics (e.g., ‘has the answers to the questions I was too afraid to ask’; Berk et al., 2013, p. 7). In the MBCT one carer became aware of their own stigmatising beliefs toward the person they support, stating: ‘Although I wanted to be understanding and supportive before... sometimes I would think oh, come on, surely you can find something that’s good in your life, it’s not really that bad.’ (Racey et al., 2018, p. 1070).

**Table 3:** Study Characteristics of Included Qualitative Studies

Study	Aims	Study quality	Design	Population	Intervention	Outcomes	Limitations
**Berk et al. (2013)**	To evaluate the acceptability and usefulness of online guidelines for caregivers of adults with bipolar.	17/20	Online feedback survey was emailed to web users. Researchers used content analysis to assess responses to open-ended questions.	Participants were adult carers of adults with bipolar.	An information website for carers of a person with bi-polar disorder.	Most caregivers positively appraised the guidelines, believing the information was supportive. A few respondents who were facing complex family problems, or who supported someone with severe, chronic bipolar were not as positive in their appraisals.	Specific to carers of a person with bipolar. Not enough information on ethics e.g., consent and confidentiality. Author didn’t critically examine role.
**Stjernswärd & Östman. (2011)**	To illuminate user’s experiences of a website aimed at helping relatives of persons with depression.	18/20	Data was collected through forum posts, a usability scale and focus groups. Analysed using content analysis.	Relatives of a person with depression.	Website access over 10 weeks and asked to contribute to online diary once per week.	Results revealed participants’ experiences of the diary and forum, advantages and obstacles to the online format and feedback on the website’s contents and use.	Small sample size. Researcher did not critically examine role.
**Racey et al. (2018)**	To assess if a parallel course for carers (parents) was a useful addition to the MBCT.	17/20	Semi-structured interviews and thematic analysis.	Young people (aged 14–18 years) with depression and their parents were recruited within a single Child and Adolescent Mental Health Service (CAMHS) in Devon.	8 session manualised group intervention.	Participants were positive about the parallel course, with comments about how it has improved their capacity as parents, benefitted the wider family, helped with understanding depression, and provided an opportunity to share with others about challenges and coping strategies. The positive responses from parents suggest the course is an acceptable intervention.	Sample lacks diversity. Researcher did not critically examine role.

#### Appropriate Intervention Design

Carers identified appropriate intervention design as a marker of intervention usefulness in all three studies. Carers made positive and negative comments about accessibility, privacy, navigation, and processes.

Participants in the two online interventions appreciated the ease of accessing information online, both within the website (e.g., ‘easy to find what I was looking for’; Berk et al., 2013, p. 7) and as an alternative to face-to-face methods (e.g., ‘convenient to use from different locations (home and work), making it easy to exploit free time’; Stjernswärd & Östman, 2011, p. 378).

Some carers regarded the online format as a more accessible option (e.g., ‘information… can be easily accessed if someone is stressed or has little time’; Berk et al., 2013). However, for others, the online format was less accessible (e.g., ‘too complex’ and ‘I felt I was being attacked by the pop-up’ and ‘too many fonts and colours on pages’; Berk et al., 2013, p. 8). There were similar positive and negative appraisals for the online social forum. Carers regarded the forum as a cheaper and more subtle therapeutic option as opposed to a psychologist and were grateful that it could be accessed at any time of day compared to in-person group meetings (Stjernswärd & Östman, 2011). Knowing that the online forum was always available when needed also gave them a feeling of security. However, despite having constant access to the website, some carers didn’t know when they would have their questions answered. Other carers felt that reading forum posts can be an unwanted reminder of difficult times, particularly during good moments when participants preferred to focus on the positives. Participants also reflected on privacy concerns and an uncomfortable feeling that they were exposing themselves and their loved ones (Stjernswärd & Östman, 2011).

For the MBCT intervention, carers were unsure about the mindfulness process. During the initial sessions, two participants described the therapy as ‘a bit odd… a bit strange’, and ‘too alternative for some’ (**Racey et al. (2018)**, p. 1069). However, one of these participants said they were eventually able to ‘understand the process’ (Racey et al., 2018, p. 1069).

### Synthesis of Quantitative and Qualitative Studies

The synthesis of qualitative and quantitative studies revealed the extent to which qualitative themes were present in quantitative studies.

All six quantitative studies referred to the theme of carer-specific needs in intervention designs through mental health promotion, social support, and relationship support. Two studies aimed to strengthen well-being skills and capacity through family-focused therapy and a self-help manual (McCann et al., 2015; Perlick et al., 2018) and interventions in four studies promoted social support through information about the benefits of connecting socially (Hubbard et al., 2016; McCann et al., 2015), stories of other carers facing similar challenges (Berk et al., 2013), and access to a private social forum (Stjernswärd & Östman, 2011). Finally, two interventions provided tangible strategies for carers to strengthen relationships with care recipients. Carers and care recipients worked together to improve CBT skills (Racey et al., 2018) and develop an action plan for managing symptoms (Hubbard et al., 2016).

Targeted psychoeducation was also mentioned in all six studies, through information about mental illness (Berk et al., 2013; Hubbard et al., 2016; Perlick et al., 2018) and supports focused on building carers’ capacity for coping (McCann et al., 2015; Racey et al., 2018; Stjernswärd & Östman, 2011).

The theme of appropriate intervention design was present in four of the six studies, through targeted content (e.g., content based on carer lived experience; see Berk et al., 2013; Hubbard et al., 2016; Perlick et al., 2018; Stjernswärd & Östman, 2011) and a consideration for accessibility limitations. Three interventions addressed accessibility in terms of physical accessibility, i.e., resources that carers could access online (Berk et al., 2013; Stjernswärd & Östman, 2011) and digestible content, i.e., the use of 'non-technical' language (Berk et al., 2013, p.3).

## Discussion

This is the first systematic review that identifies the types of supports and interventions that exist for carers of a person with depressive or anxiety symptomology and compares their suitability for this carer group. The results from this review demonstrate that while carers of a person with depressive symptomology can benefit from a variety of interventions such as, psychoeducation (Berk et al., 2013; Hubbard et al., 2016; McCann et al., 2015), family support (Perlick et al., 2018), social support (Stjernswärd & Östman, 2011) and MBCT (Racey et al., 2018), these interventions have not been systematically evaluated. The results also demonstrate a lack of suitability evidence for interventions targeting carers of a person with anxiety symptomology, indicating an important gap in the current evidence base.

### Findings

The review contributed four main findings to the area of scoping evidence-based supports for carers of a person with depressive or anxiety symptomology. The first is a summary of existing quantitative studies, with similar efficacy measures that have shown positive results for supporting this carer group.

The similarities across the interventions used in the RCTs, such as psychoeducation and health-promoting strategies, may partly explain their shared success. However, the success of unique intervention features (e.g., a take-home manual; see McCann et al., 2015) could also be due to their compatibility with distinct target groups. For instance, study participants were from different parts of the world, i.e., Perth, Australia (Perlick et al., 2018), New York, United States (Hubbard et al., 2016), and Chiang Mai, Thailand (McCann et al., 2015). Furthermore, carers in one study supported a person with a depression diagnosis (McCann et al., 2015), compared to a bipolar diagnosis in the other two studies (Hubbard et al., 2016; Perlick et al., 2018). These distinctions suggest that carers living in different geographical and social contexts or supporting someone with undiagnosed or comorbid depressive symptoms may not experience the same benefits from particular intervention features.

The research findings from the RCTs are consistent with other literature advocating for the role of psychoeducation and CBT in reducing carer strain and improving health and wellbeing for carers (Grenyer et al., 2019; Krawitz et al., 2016; Lucksted et al., 2012; Pearce et al., 2017; Takizawa et al., 2006). The mixed-methods studies included in this summary also indicated evidence for intervention feasibility, acceptability, and usefulness. These findings reflect a wider body of literature regarding the benefits of online support and face-to-face MBCT sessions for carers (Gleeson et al., 2017; Osborn et al., 2018; Spencer et al., 2019).

The second contribution was a thematic analysis of intervention suitability evidence from existing qualitative studies. Carers' positive appraisals in this analysis are consistent with other studies on interventions targeting the needs of specific carer groups (Greenwood et al., 2013; Sin et al., 2017; Sin et al 2019; Whitney et al., 2012). However, the analysis also identified drawbacks of included intervention approaches. For example, carers said they were concerned about privacy with the online social forum (Stjernswärd & Östman, 2011). Carers in other studies have made similar comments around this format (Blom et al., 2015; Dam et al., 2017). Community hesitancy around privacy online is present in other peer support research, where users discuss the importance of maintaining their anonymity (Caton et al., 2019; Chan et al., 2016; Hanna & Gough, 2018). In addition to privacy concerns, carers in the literature perceive online designs as lacking trust, quality control, and targeted information (Gibson et al., 2017). However, carers did not identify these drawbacks in this review. By contrast, the current study was in line with other evidence suggesting that the benefits of online support interventions exceed the drawbacks (Naslund et al., 2016). Furthermore, carers’ negative appraisals about the mindfulness-based therapy correspond with other evidence where carers have expressed doubts about novel or uncommon intervention designs (e.g., group singing; see Camic et al., 2013). However, like in the study by Camic et al. (2013), carers who engaged with the mindfulness techniques eventually understood and accepted the process (Racey et al., 2018).

The third finding, which compared quantitative results to qualitative themes, indicated that most quantitative studies incorporated all three qualitative themes. However, two studies (McCann et al., 2015; Racey et al., 2018) did not embed the theme of appropriate intervention design. One reason for this may be that the GSH manual and the MBCT initially aimed to support care recipients with depression rather than their carers. Nonetheless, carers' positive comments (Racey et al., 2018) and improved resilience (McCann et al., 2015) from these interventions suggest that supports designed to improve care recipients' outcomes may also benefit carers.

Finally, this paper fills a gap in the evidence base for systematically scoping interventions that exist for carers of a person with depressive or anxiety symptoms. It also identified the interventions that exist and some current gaps in the evidence base, namely, suitability evidence for supports targeting carers of a person with anxiety symptomology. It is also noteworthy that included studies typically required carer participants to be supporting someone with diagnosed depression or bipolar disorder. This finding indicates a current gap in the evidence base for supports for carers of a person with depressive or anxiety symptomology, where a formal diagnosis is not present.

### Limitations

Some limitations of the review should be taken into consideration. The studies explored within the study scope are not comprehensive in their representation of the heterogeneous needs and preferences of the carers of a person with depressive or anxiety symptomology. The included studies can only speak for small samples of carers of a person with depression or bipolar disorder exclusively. The lack of representation of carers of people with anxiety in the review suggests a need for more investigation into this subject area. It is also worth acknowledging that the results are not relevant to individuals under 16 years of age who support someone with these symptoms. Furthermore, as the search strategy did not include grey literature, the review may have excluded unpublished evidence of suitable interventions for this carer group. Thus, there is the possibility of bias toward positive outcomes of interventions, as many researchers will not publish negative findings in peer-reviewed journals.

Due to the few studies with robust evidence for intervention efficacy (three RCTs), the capacity to draw conclusions on suitable interventions for the carer group is limited. Even within these studies, sample sizes were small. There are similar limitations in using results from the qualitative studies to represent carer perspectives more broadly. Sample sizes were also small for these studies and were lacking sufficient diversity (e.g., one sample was recruited from a single Child and Adolescent Mental Health Service in Devon, England; see Racey et al., 2018). Furthermore, while illuminating, participant feedback was limited to specific intervention types, such as an online diary (Stjernswärd & Östman, 2011). More research is needed to explore carer experiences with engaging in more diverse interventions, such as those available to other carer groups.

### Conclusion and Recommendations

This review aimed to identify and assess suitability evidence for interventions targeting carers of a person with depressive or anxiety symptomology. While there was some suitability evidence for interventions targeting carers of a person with depressive symptoms, none of the included studies targeted carers of a person with anxiety symptomology specifically. Further, small sample sizes in studies meant results were not sufficiently representative of carers of a person with depressive symptomology. The qualitative analysis found that carers of a person with depressive symptoms accepted interventions that addressed their specific needs, included targeted psychoeducation, and had an appropriate intervention design. In addition, the synthesis of quantitative and qualitative studies suggested that interventions focused on improving outcomes for care recipients can also benefit carers. Despite these findings, the review indicates some gaps in suitability evidence for interventions targeting this carer group. More research is needed to understand the needs and suitable supports for these carers.

## Supplementary Materials

This systematic review is registered with the International Prospective Register of Systematic Reviews (PROSPERO, http://www.crd.york.ac.uk/PROSPERO, Registration Number = CRD42020147742) (for access see Index of Supplementary Materials below).



SawE.
Kay-LambkinF.
FitzpatrickS.
TriandafilidisZ.
McNaughtonK.
LyfordB.
HeathJ.
HardingN.
 (2020). The effectiveness of current supports and interventions for carers of people with depressive and anxiety symptoms: A systematic review.
[Study protocol]. PsychOpen. https://www.crd.york.ac.uk/prospero/display_record.php?ID=CRD42020147742
10.5964/ejop.6407PMC978073036605087
